# Exploratory Treatment-Selection Model of Intraoperative Cone-Beam Computed Tomography During Percutaneous Nephrolithotomy: Insights from RCT Data

**DOI:** 10.3390/jcm15093372

**Published:** 2026-04-28

**Authors:** Chris A. Suijker, Riemer A. Kingma, Inge M. van Oort, Stijn Roemeling

**Affiliations:** Department of Urology, University Medical Center Groningen, University of Groningen, 9713 GZ Groningen, The Netherlands; c.a.suijker@umcg.nl (C.A.S.);

**Keywords:** hybrid operating room, intraoperative cone-beam computed tomography, patient selection, percutaneous nephrolithotomy, residual fragments, urolithiasis

## Abstract

**Background/Objectives**: Intraoperative cone-beam computed tomography (CBCT) can detect residual fragments (RFs) during percutaneous nephrolithotomy (PCNL), enabling immediate removal and improving stone-free status. However, CBCT requires a hybrid operating room (OR), which is often limited in availability. This study explores patient and stone characteristics associated with CBCT eligibility and develops an exploratory treatment-selection model estimating stone-free probabilities conditional on CBCT use. **Methods**: We performed a retrospective study of a previously conducted randomized controlled trial evaluating intraoperative CBCT during PCNL in a tertiary care center. We compared CBCT-eligible cases versus ineligible cases, and cases achieving grade C (≤4 mm) stone-free status versus those with RFs. A multivariate exploratory treatment-selection model was developed using the strongest potential predictors of stone-free status. Internal validation was performed using bootstrapping. The model was also assessed for predicting grade A (0 mm) stone-free status. **Results**: The only significant difference between CBCT-eligible (*n* = 160) and ineligible (*n* = 60) cases was stone composition (*p* = 0.022). The final model included intraoperative CBCT (*p* = 0.003), stone size (*p* = 0.024), and composition (*p* = 0.044). Model-based estimates suggested smaller differences in predicted stone-free probabilities with CBCT in solitary stones. The AUC was 0.81 (95% CI: 0.73–0.88) for grade C and 0.75 for grade A (95% CI: 0.67–0.82) outcomes. Internal validation demonstrated moderate optimism, indicating potential overfitting. **Conclusions**: This exploratory treatment-selection model estimates conditional stone-free probabilities with and without CBCT. The findings suggest variation in expected benefit across stone characteristics but should be considered hypothesis-generating. The model is not intended for clinical decision-making and requires external validation before implementation.

## 1. Introduction

Intraoperative cone-beam computed tomography (CBCT) is an emerging adjunct in endourology that has the potential to improve single-session stone-free rates following percutaneous nephrolithotomy (PCNL) [1]. During percutaneous kidney stone surgery, residual fragments (RFs) are often difficult to identify within the complex renal anatomy due to their size or location. This may lead endourologists to falsely assume a stone-free status in approximately 20–50% of cases [2,3,4]. In these cases, intraoperative CBCT provides 3D imaging, enabling detection and removal of RFs missed by conventional endoscopic and fluoroscopic assessment. This is clinically important because RFs are associated with higher rates of postoperative stone-related morbidity, including persistent pain, emergency department visits, hospital admissions, drainage procedures for acute obstruction, and reinterventions aimed at fragment clearance [5].

Evidence supporting the clinical benefit of intraoperative CBCT comes from a single-center randomized controlled trial (RCT) conducted in a high-volume tertiary care center. Patients were randomized to PCNL with or without intraoperative CBCT. The trial demonstrated a 15% absolute improvement in grade C (which allows RFs ≤ 4 mm) and a 9% improvement in grade A (0 mm cutoff) stone-free rates in the CBCT group, as assessed by non-contrast CT (NCCT) 4 weeks postoperatively [6]. At 18 months of follow-up, this translated into a 10% reduction in stone-related events, including emergency department visits, hospital admissions, drainage procedures and reinterventions [7]. These findings are supported by recent cohort studies reporting superior stone-free rates in patients undergoing PCNL with intraoperative CBCT [8,9,10], underscoring the potential of this technology to improve PCNL outcomes.

Although intraoperative CBCT improves stone-free rates and reduces stone-related events, routine use has limitations. Imaging may prolong operative time and increase radiation exposure. In addition, CBCT requires a hybrid operating room (OR) equipped with dedicated imaging technology. Such rooms are costly and often limited in availability due to competing demands from other specialties [6,8,9,10]. Efficient utilization of hybrid OR capacity therefore requires careful patient selection. Certain patients may derive limited benefit from intraoperative CBCT, either because RFs are already clearly visible on conventional fluoroscopy or endoscopy, making CBCT redundant, or because additional imaging provides minimal incremental benefit for further stone extraction. Identifying such cases is essential to ensure judicious use of radiation and scarce and expensive hybrid OR resources.

Accordingly, strategic prioritization of patients most likely to benefit from intraoperative CBCT may optimize both clinical outcomes and resource utilization. However, it remains unclear which patient or stone characteristics are associated with differential benefit from intraoperative CBCT, and whether such information can support allocation of limited hybrid operating room capacity. Existing PCNL scoring systems primarily estimate procedural complexity or the probability of stone-free status, but do not directly address the incremental value of intraoperative CBCT.

The aim of this study is to retrospectively explore patient- and stone-related characteristics associated with CBCT eligibility and benefit, using data from the aforementioned RCT. Rather than developing a purely prognostic model, we will construct an exploratory treatment-selection model estimating stone-free probabilities conditional on CBCT use. This allows the visualization of potential differences in outcomes across patient subgroups. The model is intended as hypothesis-generating, may include variables not available preoperatively, and is therefore not yet suitable as a preoperative decision tool or for clinical decision-making.

## 2. Materials and Methods

This retrospective study is based on the previously conducted single-center, parallel, two-arm, non-blinded RCT, named CAPTURE. This trial was conducted between January 2020 and July 2023 at the University Medical Center Groningen, The Netherlands, a tertiary referral center for kidney stone treatment. The original RCT and this study were conducted in accordance with the Declaration of Helsinki, and approved by the Medical Ethics Review Committee of the University Medical Center Groningen on 12 December 2019 (approval no. 2019/375). Written informed consent was obtained from all patients. Data collection and the study were monitored by an independent monitor. No external funding was received. The methodology of the original RCT is summarized below, followed by the design of the current study.

### 2.1. Original RCT Design

In the CAPTURE RCT, the primary objective was to investigate whether intraoperative CBCT could detect additional RFs and allow their extraction in patients deemed stone-free by conventional endoscopy and fluoroscopy. All adult (≥18 years), competent, non-pregnant patients with urolithiasis suitable for PCNL treatment were approached for inclusion.

Intraoperatively, patients who were enrolled but could not achieve a fluoroscopic or endoscopic stone-free status, or who experienced complications that rendered CBCT images unreliable, were not randomized. Performing intraoperative CBCT in these cases was unlikely to provide added value. Consequently, randomization was restricted to cases considered stone-free by conventional endoscopy and fluoroscopy, meaning that eligibility for CBCT was determined intraoperatively rather than preoperatively.

Randomization was performed in a 1:1 ratio using block randomization with randomly sequenced blocks of varying sizes and was implemented electronically via the Research Electronic Data Capture (REDCap) randomization module. Patients assigned to the CBCT group underwent intraoperative CBCT to identify potential RFs, and surgery continued to extract any detected fragments. In the control group, the procedure was concluded without CBCT. The primary outcome was the difference in grade C (≤4 mm) stone-free rate on 4-week postoperative non-contrast CT (NCCT) between the CBCT and control groups.

#### 2.1.1. Surgical Procedures

All procedures were performed by a single surgeon with extensive experience (>10 years) in endoscopic stone surgery (S.R.). Percutaneous puncture was obtained under combined fluoroscopic and ultrasound guidance, without CBCT. Surgical aspects such as patient positioning, tract size, tract number, fragmentation techniques, and use of endoscopic combined intrarenal surgery (ECIRS) were not standardized. Access tract diameters ranged from ultra-mini (outer diameter 13 F) to mini (outer diameter 17.5 F) and full-size (outer diameter 24 F). Stones were fragmented with either laser lithotripsy or a combination of pneumatic and ultrasonic lithotripsy. Stones were evacuated with grasping forceps or flushing techniques. Patients with bilateral stones undergoing staged PCNL were randomized separately for each procedure. Study flow diagrams of the CAPTURE-RCT have been published previously [6,7].

#### 2.1.2. Cone-Beam Computed Tomography Protocol

All procedures were conducted in a hybrid OR with a radiolucent carbon fiber operating table and an ArtisQ Ceiling DynaCT (Siemens Healthineers, Erlangen, Germany), which functions both as a flat-panel fluoroscopy detector and as a CBCT scanner. At our center, the urology-specific hybrid OR is available two days per week for procedures requiring general anesthesia. The CBCT acquisition protocol was based on a prior phantom study and consisted of a 6 s scan with 397 frames, delivering a radiation dose of 0.08–0.36 µGy per frame.

In patients randomized to the CBCT-group, the nephroscopy sheath was removed prior to image acquisition to reduce scatter artifacts. Guidewires were left in situ to allow rapid reinsertion of the sheath if required. Patient positioning remained unchanged. However, a test rotation was performed to ensure safe rotation without collision risk. During CBCT image acquisition, a brief apnea was induced by the anesthesiologist to minimize motion artifacts. The CBCT scanner and its control panel were covered in a sterile manner, allowing the urologist to control the scanner intraoperatively.

#### 2.1.3. Power and Interim Analysis

Sample size calculations were based on a prior feasibility study, assuming an absolute difference of 15% in grade C (≤4 mm) stone-free rates on 4-week postoperative NCCT. With a power of 80% and an *α* value of 0.05, the required sample size was estimated at 152 cases per group. Allowing for a 5% safety margin, the target enrollment was set at 320 randomized cases. Data were also collected from enrolled patients who could not be randomized due to intraoperative complications or failure to achieve an endoscopic and fluoroscopic stone-free status.

A predefined interim analysis was predefined at 160 randomizations. The trial was discontinued prematurely after meeting the predefined superiority criterion, defined as a statistically significant difference exceeding 15% in grade C (≤4 mm) stone-free rates in favor of the CBCT group.

### 2.2. Current Study Design

The current analysis included all participants from the original trial. Data were categorized into three groups: (1) cases ineligible for intraoperative CBCT due to complications or persistent endoscopic/fluoroscopic RFs, (2) randomized cases with grade C (≤4 mm) stone-free status at 4-week postoperative NCCT, and (3) randomized cases with RFs > 4 mm at 4-week postoperative NCCT.

Group 1 was compared with groups 2 and 3 combined using univariate analysis to identify baseline differences between CBCT-eligible and ineligible patients. This analysis was performed to explore factors associated with intraoperative CBCT eligibility, recognizing that eligibility was determined intraoperatively after conventional fluoroscopic and endoscopic assessment. Potential preoperative predictors of CBCT eligibility were assessed, including age, sex, American Society of Anesthesiologists (ASA) class, procedure side, body mass index (BMI), abdominal circumference, skin-to-stone distance, prior stone treatment (<1 year), anatomical abnormalities (horseshoe kidney, urinary tract deviations, kidney transplant, UPJ stenosis), neurological abnormalities (spina bifida, cerebral palsy, spinal injury), preoperative hydronephrosis, preoperative drainage, and stone characteristics (maximum essence, volume, size, location, composition).

In a second analysis, groups 2 and 3 were compared to identify potential predictors of stone-free status at 4 weeks postoperatively. The same set of preoperative variables was analyzed univariately, with intraoperative CBCT included as an additional variable. Variables with *p* < 0.157 in univariate analysis were considered for multivariate binary logistic regression. This threshold follows purposeful selection recommendations to avoid excluding potentially important variables [11,12]. Because intraoperative CBCT is a treatment variable rather than a baseline predictor, the model estimates outcomes conditional on treatment rather than providing purely prognostic predictions. As the analysis is based on RCT data, the treatment variable of intraoperative CBCT could be included as a predictor without concern, as previous analyses demonstrated no baseline differences between the randomized groups [6,13]. Formal treatment–predictor interaction terms were not included due to limited sample size; therefore, subgroup differences should be interpreted as exploratory. Based on regression results, an exploratory treatment-selection model was developed. Predicted model-based probabilities were calculated to visualize conditional outcome probabilities with and without CBCT, and model performance was evaluated for both grade C (≤4 mm) and grade A (0 mm) stone-free status.

### 2.3. Data Processing and Statistical Analysis

All data were handled in accordance with Good Clinical Practice (GCP) and the General Data Protection Regulation (GDPR). Statistical analyses were performed using R Studio version 2024.12.0+467 (R Foundation for Statistical Computing, Vienna, Austria). Maximum Hounsfield unit (HU) values, used to determine stone composition, as well as stone volume, were measured from CT scans using automatic density assessments in Aquarius Intuition version 4.10.2.17-47 (TeraRecon Inc., Durham, NC, USA). In this software, intrarenal structures with HU > 350 were automatically segmented, and their volume was calculated. ASA class was determined from anesthesiology screening, and stone composition was assessed using postoperative stone laboratory analysis.

Stone composition was divided into four categories based on the predominant component: calcium oxalate, infectious stones, uric acid stones, and other or unknown stones. Infectious stones were defined as stones composed of struvite (magnesium ammonium phosphate) and/or carbonate apatite, typically associated with urease-producing bacterial infections. The category “other or unknown stones” included all remaining stone types, such as cystine stones, brushite (calcium phosphate), and mixed compositions not classified in the aforementioned groups, as well as cases in which stone composition analysis was unavailable.

Abdominal circumference was measured from CT imaging at the level of the umbilicus in Aquarius Intuition. Skin-to-stone distance was measured manually from CT imaging by drawing two perpendicular lines from the skin and a third line at a 45° angle relative to these lines, which represented the skin-to-stone distance. Other variables were obtained from patients’ medical histories and from CT scans stored in the electronic health records.

For univariate analysis, Mann–Whitney U tests or *t*-tests were used for continuous variables, depending on distribution. Chi-squared tests were used for categorical variables. Patients undergoing staged bilateral PCNL were treated as independent events in the analysis. Because procedures performed in the same patient may share biological and clinical characteristics, this approach may violate the independence assumption of standard logistic regression and underestimate variance. Given the limited number of bilateral cases, clustering was not modelled, and this was addressed as a limitation.

Final variable selection for development of the multivariate logistic regression model considered performance, multicollinearity, clinical relevance, and model parsimony. Variables with missing values <5% were analyzed using complete-case analysis with exclusion of individuals with missing values; no variable had >5% missing data. Sensitivity analyses using multiple imputation ensured that missing data did not materially affect model outcomes. Multicollinearity was assessed using variance inflation factors (VIF), with VIF > 5 considered indicative of high collinearity. Linearity of continuous predictors with the log-odds was evaluated as part of model development.

Model goodness-of-fit was evaluated using the Hosmer–Lemeshow test, discrimination using the area under the receiver operating characteristic curve (AUC), and calibration by comparing predicted and observed outcomes and using calibration curves. Internal validation was performed via bootstrapping with 1000 resamples, yielding optimism-corrected AUC and bias-corrected calibration curves. Given the limited number of outcome events, the model was considered exploratory and potentially prone to overfitting. Regression coefficients from the final model were used to estimate predicted probabilities of stone-free outcomes across subgroups.

This study was conducted and reported in accordance with the TRIPOD (Transparent Reporting of a multivariable prediction model for Individual Prognosis Or Diagnosis) reporting guidelines. This analysis is exploratory and hypothesis-generating and is not intended to define definitive clinical decision thresholds suitable for clinical decision-making.

## 3. Results

Between January 2020 and July 2023, 222 cases were included in the CAPTURE-RCT, of which 220 underwent percutaneous kidney stone treatment. Of these, 60 cases (27%) were ineligible for randomization, and 160 cases (73%) were randomized. Among the 60 CBCT-ineligible cases, the most common reasons for ineligibility were failure to achieve an intraoperative endoscopic stone-free status within the operative time limit (27 cases, 45%) and intraoperative hemorrhage (8 cases, 13%). Of the randomized cases, 155 underwent a 4-week NCCT to assess the primary outcome. The 5 cases with missing 4-week postoperative NCCT scans due to patient no-shows were excluded, in accordance with complete-case analysis. A small number of patients (*n* = 11) underwent staged bilateral procedures, which were analyzed as independent observations. Based on a grade C (≤4 mm) stone-free definition, 110 (71%) cases were stone-free, and 45 (29%) had RFs. Using a stricter grade A (0 mm) stone-free definition, 73 (47%) cases were stone-free, and 82 (53%) had RFs.

Table 1 presents the differences in baseline characteristics between CBCT-eligible and CBCT-ineligible patients. No statistically significant differences in preoperative characteristics were observed, except for dominant stone composition (*p* = 0.022). Patients eligible for intraoperative CBCT more frequently had calcium oxalate stones (51% vs. 33%), whereas ineligible patients more often had unknown or atypical stone compositions (25% vs. 11%). Stone volume tended to be lower in the CBCT-eligible group, with a median volume of 450 mm^3^ (IQR: 160–1165) compared to 720 mm^3^ (IQR: 278–2250) in the CBCT-ineligible group. This difference did not reach statistical significance (*p* = 0.052).

Table 2 shows the differences in characteristics between cases that achieved a grade C (≤4 mm) stone-free status after randomization and those who did not. Statistically significant differences were observed for stone volume (*p* = 0.015), use of intraoperative CBCT (*p* = 0.016), stone size (*p* < 0.001), and stone composition (*p* = 0.003). Single stones < 20 mm, intraoperative CBCT use, and lower stone volumes were associated with stone-free outcomes, whereas infectious stones were more frequently observed among patients with RFs.

Although differences in prior treatment (*p* = 0.103), stone essence (*p* = 0.105), and stone location (*p* = 0.051) did not reach conventional statistical significance, they met the predefined threshold (*p* < 0.157) for inclusion in multivariable regression analysis. Of the 7 candidate variables, 3 were retained in the final multivariate binary logistic regression model: intraoperative CBCT use, stone size, and stone composition. No variables were excluded due to multicollinearity (VIF > 5). There were no missing values for the variables included in the final model. The results of the multivariate analysis are presented in Table 3 and the full model equation for predicting grade C (≤4 mm) stone-free rates is presented in Appendix A.

Several predictors, particularly less prevalent stone compositions such as uric acid stones, demonstrated wide confidence intervals, reflecting limited subgroup sizes and resulting uncertainty in the corresponding effect estimates. Univariate differences in characteristics between cases achieving grade A (0 mm) stone-free status and those with RFs are presented in Appendix B, Table A1, alongside the final variable selection for multivariate analysis applied to grade A stone-free status (Appendix B, Table A2). The full model equation for predicting grade A (0 mm) stone-free rates is presented in Appendix C.

**Figure 1:**
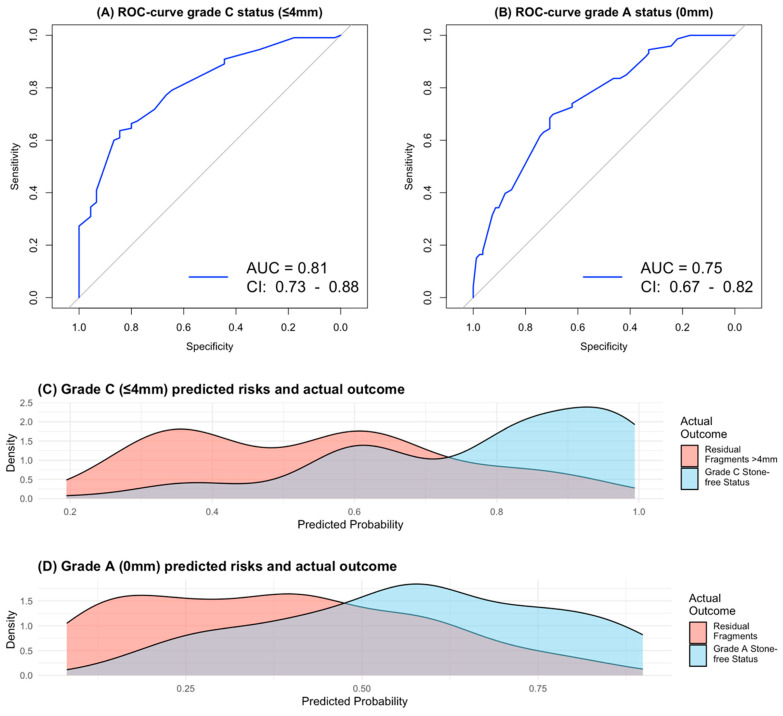
(**A**) ROC curve of the prediction model predicting grade C (≤4 mm) stone-free status after percutaneous nephrolithotomy. (**B**) ROC curve of the prediction model predicting grade A (0 mm) stone-free status after PCNL. (**C**) Comparison of predicted probability and actual grade C stone-free outcome in the original dataset. (**D**) Comparison of predicted probability and actual grade A stone-free outcome in the original dataset. AUC = area under the curve; CI = confidence interval; ROC = receiver operating characteristic; PCNL = percutaneous nephrolithotomy.

The ROC curve for predicting grade C (≤4 mm) stone-free status demonstrated strong discriminatory performance, with an area under the curve (AUC) of 0.81 (95% CI: 0.73–0.88) (Figure 1A). After internal validation using 1000 bootstrap resamples, the optimism-corrected AUC was 0.76 (95% CI: 0.69–0.83), corresponding to a 5% reduction in performance and indicating moderate overfitting. The Hosmer–Lemeshow test suggested good model fit (*p* = 0.964).

For prediction of grade A (0 mm) stone-free status, the ROC curve yielded an AUC of 0.75 (95% CI: 0.67–0.82), with an optimism-corrected AUC of 0.73 (95% CI: 0.70–0.75) following internal validation (Figure 1B). The Hosmer–Lemeshow test again indicated adequate model fit (*p* = 0.476). Figure 1C illustrates that predicted probabilities in comparison to actual outcomes for grade C stone-free status were generally consistent. In contrast, Figure 1D demonstrates reduced correspondence between predicted probabilities and observed outcomes for a grade A stone-free status.

**Figure 2:**
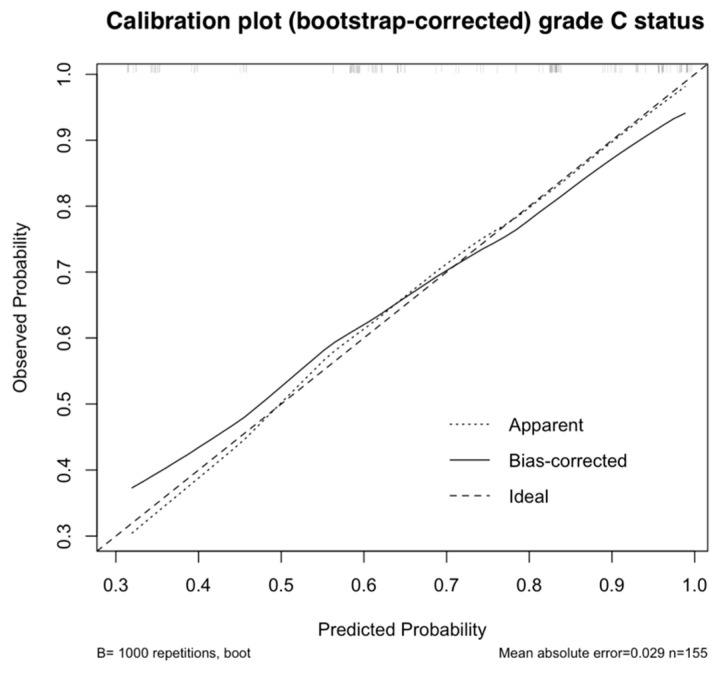
Apparent and bias-corrected (after 1000 bootstrap samples) calibration plot of model performance when predicting grade C (≤4 mm) stone-free status.

**Figure A1:**
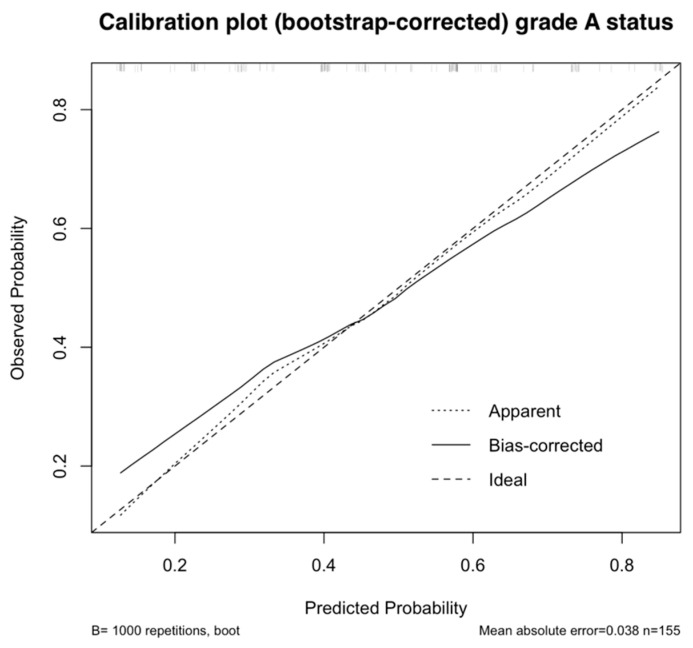
Apparent and bias-corrected (after 1000 bootstrap samples) calibration plot of model performance when predicting grade A (0 mm) stone-free status.

The calibration curve for predicting grade C (≤4 mm) stone-free status is presented in Figure 2, whereas the calibration curve for predicting complete (0 mm) stone-free status is shown in Appendix D, Figure A1. Although calibration was acceptable in the original dataset, bias-corrected results demonstrated underestimation of risk at lower predicted probabilities and overestimation at higher probabilities. These calibration issues were more pronounced for prediction of grade A (0 mm) stone-free status.

**Figure 3:**
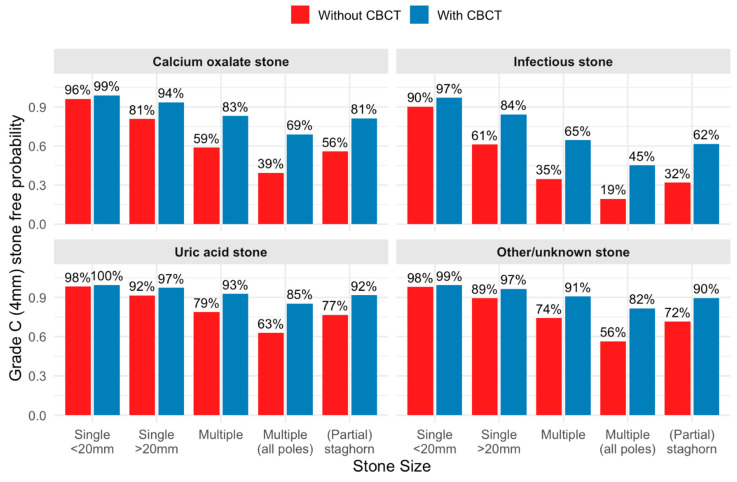
Predicted probabilities of achieving grade C (≤4 mm) stone-free status on 4-week non-contrast computed tomography (NCCT), based on the three variables included in the exploratory multivariate model: stone composition, stone size and use of intraoperative CBCT. CBCT = cone-beam computed tomography.

**Figure A2:**
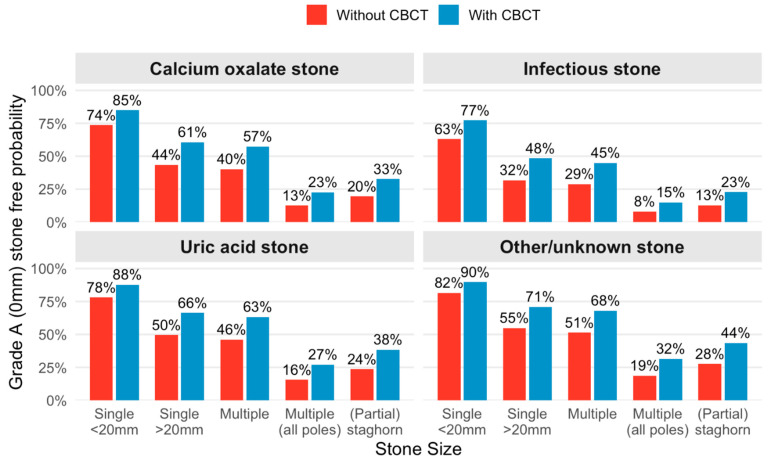
Predicted probabilities of achieving grade A (0 mm) stone-free status on 4-week non-contrast computed tomography (NCCT), based on the three variables included in the exploratory multivariate model: stone composition, stone size and use of intraoperative CBCT. CBCT = cone-beam computed tomography.

Predicted probabilities of achieving a grade C (≤4 mm) stone-free status, based on the three variables included in the final multivariable model, are presented in Figure 3. Patients with single stones < 20 mm exhibited high predicted stone-free probabilities (90–100%), resulting in small model-based differences between predictions with and without CBCT. As stone complexity increased, these model-based differences became larger, although no formal interaction analysis was performed. Notably, multiple stones involving all renal poles were associated with poorer predicted outcomes than partial or complete staghorn stones. Uric acid stones showed the highest predicted stone-free rates, with model-based differences exceeding 15% only in more complex cases involving multiple stones across all renal poles. In contrast, infectious stones were associated with the lowest predicted probabilities of achieving a stone-free outcome. Corresponding predicted model-based probabilities for achieving a grade A (0 mm) stone-free status are shown in Appendix D, Figure A2.

## 4. Discussion

This exploratory treatment-selection analysis aimed to identify which patient profiles may derive greater benefit from intraoperative CBCT during PCNL. Among 160 CBCT-eligible and 60 ineligible cases, stone composition was the only significant baseline difference. However, when comparing 110 cases with a grade C (≤4 mm) stone-free status to 45 cases with RFs > 4 mm, significant univariate differences emerged in stone volume, stone size, stone composition, and CBCT use. The latter three variables were incorporated into an exploratory model estimating stone-free probabilities conditional on CBCT use, allowing calculation and visualization of predicted improvements in stone-free rates with and without intraoperative CBCT across different patient populations.

Importantly, this model should not be interpreted as a purely prognostic tool, but rather as an exploratory framework to evaluate potential heterogeneity in treatment effect. To our knowledge, this is the first study to integrate intraoperative CBCT into a treatment-selection model for PCNL outcomes. Because CBCT represents an intervention rather than a baseline predictor, the model estimates conditional outcomes rather than identifying purely preoperative risk factors. Our findings suggest that the impact of CBCT increases with greater stone complexity and that CBCT may be particularly beneficial in calcium oxalate and infectious stones. However, formal interaction testing was not performed, so these observations reflect differences in baseline risk rather than proven subgroup-specific treatment effects. It is important to note that these findings illustrate potential heterogeneity but do not establish treatment-effect modification. Additionally, no clear preoperative clinical markers could predict CBCT ineligibility a priori, limiting definitive recommendations. As the model was developed only in cases that were intraoperatively eligible for CBCT and includes variables generally not available preoperatively, it does not represent the full preoperative decision pathway required for hybrid operating room allocation.

Accordingly, the present analysis should be viewed as a conceptual step toward treatment selection rather than a ready preoperative triage model. Our model-derived probabilities indicate potential limited benefit of CBCT for achieving grade C (≤4 mm) stone-free status in cases with single stones < 20 mm, and only marginal (<10%) benefit in larger (>20 mm) but non-calcium-oxalate and non-infectious solitary stones. These estimates should be interpreted cautiously given the limited sample size and wide confidence intervals. If externally validated and combined with a model predicting intraoperative CBCT eligibility, such an approach may support selective use of hybrid PCNL by identifying patient groups in whom intraoperative CBCT is unlikely to meaningfully improve stone-free outcomes, thereby improving allocation of limited hybrid operating room resources.

Our findings align with previous exploratory work suggesting that intraoperative CBCT adds value primarily in more complex kidney stone cases. One earlier study linked a higher Guy’s stone score to increased detection of RFs and subsequent extraction following intraoperative CBCT [14]. Although we did not apply the Guy’s stone score directly, we captured its individual components (stone size, anatomical complexity, and neurological abnormalities) to preserve granularity.

Despite the modest sample size, our exploratory model shares similarities with existing literature. Stone-free rates decrease with increasing stone burden, particularly in (partial) staghorn stones or cases with multiple stones across renal locations, consistent with prior studies identifying prognostic factors [15,16,17,18,19,20]. Unlike many existing models, ours included stone composition, which demonstrated associative relevance in our study population and may be inferred preoperatively from prior stone analyses, metabolic abnormalities, or enhanced CT imaging such as dual energy CT. However, stone composition was determined using postoperative laboratory analysis, and therefore was not uniformly available at the time of surgical planning. This limits the model’s direct preoperative applicability, underscores its exploratory nature, and complicates its eventual clinical implementation.

Conversely, our exploratory model did not retain factors such as prior endoscopic treatment, anatomical or neurological abnormalities, preoperative hydronephrosis, or stone location, despite their inclusion in other studies [15,16,17,18,19,20]. This may reflect our variable selection strategy or the specific context of our tertiary referral center, where PCNL is routinely performed in complex, comorbid patients using advanced techniques. In addition, predictor selection relied on univariable screening, which may have contributed to instability in variable inclusion and should be interpreted cautiously. It may also relate to differences in endpoint definition, as many studies rely on plain radiography or immediate postoperative imaging, whereas we used NCCT at 4 weeks postoperatively, potentially leading to different outcome classifications.

Among our cases, patients with multiple stones distributed across all renal poles demonstrated the lowest stone-free rates. This counterintuitive finding likely reflects technical rather than volumetric complexity, as dispersed calculi are more difficult to locate and fragment than a more clustered (partial) staghorn configuration. Although stone volume differed significantly between outcome groups, it was excluded from the final model in favor of stone size, which showed stronger predictive performance and greater clinical applicability. Stone location demonstrated a borderline significant association with outcomes: RFs > 4 mm were more frequent in cases with stones across multiple locations, whereas stone-free status was more commonly achieved in patients with predominantly pelvic or lower pole stone burden. However, its predictive value was insufficient to justify inclusion in the final model.

This study has several limitations. First, it was based on single-center, single-surgeon data from a tertiary referral center, which limits the generalizability of the findings to institutions with different case mixes, surgical expertise, or resource availability. As a model-based exploratory analysis, the results are hypothesis-generating, particularly given the relatively small sample size, with only 45 cases with RFs > 4 mm compared to 110 stone-free cases. Several predictors, especially less prevalent stone compositions such as uric acid stones, were associated with wide confidence intervals, reflecting unstable effect estimates due to small subgroup sizes. Consequently, predicted probabilities for these subgroups should be interpreted as indicative trends rather than precise estimates. In addition, the parent randomized trial was stopped early after interim analysis, which may have contributed to overestimation of treatment effects and further limits model stability. Nevertheless, to our knowledge, larger datasets on PCNL that report on intraoperative CBCT remain scarce, particularly those derived from randomized controlled trial designs that allow modeling of treatment-related variables [13,21].

Although the model demonstrated good initial discrimination (AUC 0.81, 95% CI: 0.73–0.88), performance declined by 5% following internal validation with bootstrapping, and the bias-corrected calibration curve indicated evidence of overfitting. These findings, together with the limited number of events, suggest that the model is statistically fragile and requires external validation before clinical application. Future studies with larger datasets may additionally consider shrinkage techniques or penalized regression methods to improve model stability and reduce overfitting.

Additionally, the findings have limited generalizability beyond our tertiary referral population, which typically includes patients with higher comorbidity and recurrence rates [7]. Moreover, intraoperative factors such as patient positioning, tract size, and use of ECIRS were excluded, as these are determined dynamically during surgery and are difficult to incorporate into decision-making. In addition, bilateral procedures were analyzed at the case level and treated as independent observations, which may have led to underestimation of variance due to within-patient correlation, potentially inflating the precision of effect estimates.

We used a grade C (≤4 mm) cutoff in model construction to align with the primary endpoint of the randomized trial. However, growing evidence suggests that stricter definitions, such as grade A (0 mm) stone-free status, may better reflect clinically meaningful outcomes, as even small RFs are associated with increased morbidity and reintervention rates [22,23,24,25]. Accordingly, we also assessed model performance for predicting grade A (0 mm) stone-free status.

Our findings suggest that the impact of intraoperative CBCT on achieving grade A (0 mm) stone-free status is greater in patients with lower stone complexity and smaller in those with multiple stones across all poles or (partial) staghorn stones (Appendix D, Figure A2), compared with grade C (≤4 mm) stone-free status. This likely reflects the inherent difficulty of completely clearing high stone burden cases. Future well-powered studies in diverse populations are needed to confirm whether these exploratory associations remain when stricter stone-free definitions are used. Importantly, the model does not provide deterministic thresholds for CBCT use and should not be used for clinical decision-making. Assessment of clinical utility, including decision curve analysis and exploration of potential clinical thresholds, should be conducted in future externally validated studies.

Finally, while our model predicts radiological outcomes, it does not address direct clinically relevant endpoints such as quality of life or prevention of stone-related events, including persistent pain, emergency department visits, hospital admissions, drainage procedures, or retreatment. These factors are essential when optimizing hybrid operating room allocation and should be incorporated into future studies. In addition, decision making should also incorporate operating time, costs, radiation exposure and organizational constraints. Future research must therefore incorporate patient-centered outcomes, evaluate clinical utility, and externally validate the model in diverse clinical settings with larger patient populations. Nevertheless, our findings represent an initial exploratory step toward optimizing the use of CBCT and hybrid OR environments in endourology and urolithiasis management.

## 5. Conclusions

Selecting patients for hybrid PCNL with intraoperative CBCT remains challenging. In this exploratory treatment-selection analysis, a model based on stone size, stone composition, and CBCT use suggested that intraoperative CBCT may be associated with higher stone-free rates, particularly in complex calcium oxalate or infectious stones, while offering limited added value in patients with solitary kidney stones. However, the model includes variables not consistently available preoperatively, was developed on intraoperatively eligible cases only, and is based on a limited number of events. These findings should therefore not be interpreted as evidence of treatment-effect heterogeneity but rather as exploratory model-based estimates. Accordingly, the model is intended as hypothesis-generating rather than a ready clinical decision tool.

Future work should combine prediction of intraoperative CBCT eligibility with treatment-effect modelling, incorporate clinically relevant outcomes, and externally validate the model in larger multicenter cohorts. Despite these limitations, the present analysis provides a conceptual framework for studying targeted intraoperative CBCT use during PCNL and may inform future efforts to optimize allocation of limited hybrid operating room resources.

## Figures and Tables

**Table 1:** Comparison of characteristics between cases eligible for intraoperative CBCT versus cases ineligible for intraoperative CBCT.

Variable	Eligible for CBCT (*n* = 160)	Not Eligible for CBCT (*n* = 60)	*p*-Value
Baseline characteristics			
Age at inclusion, years, median (IQR)	57 (48–67)	59 (44–69)	0.93 *
Female sex, *n* (%)	69 (43)	32 (53)	0.176 **
ASA class 3 or 4, *n* (%)	58 (36)	20 (33)	0.69 **
Left procedure side, *n* (%)	86 (53)	33 (55)	0.87 **
Weight characteristics			
BMI, kg/m^2^, median (IQR)	27.2 (24.1–31.1)	28.0 (24.8–32.4)	0.33 *
Abdominal circumference, cm, median (IQR)	99 (92–109)	104 (89–116)	0.31 *
Skin-to-stone distance, cm, median (IQR)	106 (89–119)	102 (89–125)	0.99 *
Preoperative profile			
Treatment < 1 year prior to PCNL, *n* (%)	40 (25)	18 (30)	0.45 **
Anatomical abnormalities, *n* (%)	36 (23)	19 (32)	0.162 **
Neurological abnormalities, *n* (%)	15 (9)	6 (10)	0.89 **
Preoperative hydronephrosis, *n* (%)	36 (23)	11 (18)	0.50 **
Preoperative drainage, *n* (%)	35 (22)	9 (15)	0.256 **
Radiological characteristics			
Maximum stone essence, HU, median (IQR)	1214 (969–1533)	1186 (910–1426)	0.48 *
Stone volume, mm^3^, median (IQR)	450 (160–1165)	720 (278–2250)	0.052 *
Stone size			0.48 **
Single stone < 20 mm, *n* (%)	32 (20)	9 (15)	
Single stone > 20 mm, *n* (%)	12 (8)	5 (8)	
Multiple stones in ≤2 poles, *n* (%)	77 (48)	25 (42)	
Multiple stones in 3 poles, *n* (%)	15 (9)	6 (10)	
Partial or complete staghorn stone, *n* (%)	24 (15)	15 (25)	
Stone location			0.85 **
Pelvis, *n* (%)	22 (14)	6 (10)	
Upper pole, *n* (%)	6 (4)	3 (5)	
Middle pole, *n* (%)	5 (3)	2 (3)	
Lower pole, *n* (%)	50 (31)	16 (27)	
Multiple sites, *n* (%)	77 (48)	33 (55)	
Dominant stone composition			0.022 **
Calcium oxalate, *n* (%)	82 (51)	20 (33)	
Infectious, *n* (%)	51 (32)	22 (37)	
Uric Acid, *n* (%)	10 (6)	3 (5)	
Other or unknown composition, *n* (%)	17 (11)	15 (25)	

ASA = American Society of Anesthesiologists; BMI = body mass index; CBCT = cone-beam computed tomography; HU = maximum Hounsfield units; IQR = interquartile range; PCNL = percutaneous nephrolithotomy. * Mann–Whitney U Test, ** Chi-squared test.

**Table 2:** Comparison of characteristics between grade C (≤4 mm) stone-free cases after randomization versus cases with residual fragments > 4 mm after randomization.

Variable	Stone-Free, Grade C (*n* = 110)	Residual Fragments > 4 mm (*n* = 45)	*p*-Value
Baseline characteristics			
Age at inclusion, years, median (IQR)	57 (47–65)	58 (48–68)	0.44 *
Female sex, *n* (%)	46 (42)	20 (44)	0.76 **
ASA class 3 or 4, *n* (%)	40 (36)	14 (31)	0.53 **
Left procedure side, *n* (%)	56 (51)	25 (56)	0.60 **
Weight characteristics			
BMI, kg/m^2^, median (IQR)	27.3 (24.2–30.9)	26.5 (23.6–31.2)	0.57 *
Abdominal circumference, cm, median (IQR)	99 (91–111)	98 (92–106)	0.57 *
Skin-to-stone distance, cm, median (IQR)	107 (89–121)	105 (89–117)	0.31 *
Preoperative profile			
Treatment < 1 year prior to PCNL, *n* (%)	23 (21)	15 (33)	0.103 **
Anatomical abnormalities, *n* (%)	27 (25)	9 (20)	0.54 **
Neurological abnormalities, *n* (%)	10 (9)	5 (11)	0.70 **
Preoperative hydronephrosis, *n* (%)	25 (23)	11 (24)	0.82 **
Preoperative drainage, *n* (%)	21 (19)	12 (27)	0.296 **
Radiological characteristics			
Maximum stone essence, HU, median (IQR)	1273 (1005–1594)	1103 (900–1425)	0.105 *
Stone volume, mm^3^, median (IQR)	390 (158–920)	910 (180–2665)	0.015 *
Intraoperative CBCT, *n* (%)	60 (55)	15 (33)	0.016 **
Stone size			<0.001 **
Single stone < 20 mm, *n* (%)	31 (28)	1 (2)	
Single stone > 20 mm, *n* (%)	10 (9)	2 (4)	
Multiple stones in ≤2 poles, *n* (%)	50 (45)	24 (53)	
Multiple stones in 3 poles, *n* (%)	6 (6)	8 (18)	
Partial or complete staghorn stone, *n* (%)	12 (12)	10 (22)	
**Stone location**			0.051 **
Pelvis, *n* (%)	17 (16)	4 (9)	
Upper pole, *n* (%)	5 (5)	1 (2)	
Middle pole, *n* (%)	5 (5)	0 (0)	
Lower pole, *n* (%)	38 (35)	10 (22)	
Multiple sites, *n* (%)	45 (41)	30 (67)	
**Dominant stone composition**			0.003 **
Calcium oxalate, *n* (%)	62 (56)	18 (40)	
Infectious, *n* (%)	26 (24)	24 (53)	
Uric Acid, *n* (%)	8 (7)	1 (2)	
Other or unknown composition, *n* (%)	14 (13)	2 (4)	

ASA = American Society of Anesthesiologists; BMI = body mass index; CBCT = cone-beam computed tomography; HU = Hounsfield units; IQR = interquartile range; PCNL = percutaneous nephrolithotomy. * Mann–Whitney U Test, ** Chi-squared test.

**Table 3:** Final multivariate binary logistic regression results of predictors for a grade C (≤4 mm) stone-free status after percutaneous nephrolithotomy.

Variable	Model Coefficients	Odds Ratio	95% CI	*p*-Value
Intercept	3.22			
Single stone < 20 mm (reference class)				0.024
Single stone > 20 mm	−1.78	0.17	0.01–2.33	0.184
Multiple stones in ≤2 poles	−2.86	0.06	0.01–0.47	0.007
Multiple stones in 3 poles	−3.65	0.03	<0.01–0.26	0.002
Partial or complete staghorn stone	−2.98	0.05	0.01–0.48	0.009
Calcium oxalate stone (reference class)				0.044
Infectious stone	−0.99	0.37	0.15–0.92	0.032
Uric acid stone	0.96	2.60	0.28–24.57	0.405
Other or unknown stone composition	0.69	1.99	0.36–10.99	0.429
No intraoperative CBCT (reference class)				0.003
Intraoperative CBCT	1.23	3.41	1.50–7.78	0.003

CBCT = cone-beam computed tomography; CI = confidence interval.

## Data Availability

The data supporting the findings of this study are not publicly available due to privacy and ethical restrictions imposed by the institution but may be made available by the corresponding author upon reasonable request.

## Abbreviations

The following abbreviations are used in this manuscript:

ASA	American Society of Anesthesiologists
AUC	Area under the receiver operating characteristic curve
BMI	Body mass index
CBCT	Cone-beam computed tomography
GCP	Good clinical practice
GDPR	General data protection regulation
NCCT	Non-contrast computed tomography
OR	Operating room
PCNL	Percutaneous nephrolithotomy
RCT	Randomized controlled trial
REDCap	Research electronic data capture
RFs	Residual fragments
ROC	Receiver operating characteristic
UPJ	Uteropelvic junction
VIF	Variance inflation factors

## Appendix A

Full Model Equation. The predicted probability of the outcome (grade C ≤ 4 mm stone-free rate) can be calculated using a logistic regression model defined as: logit(p) = 3.22 − 1.78 × (single stone > 20 mm) − 2.86 × (multiple stones in ≤2 poles) − 3.65 × (multiple stones in 3 poles) − 2.98 × (partial or complete staghorn stone) − 0.99 × (infectious stone) + 0.96 × (uric acid stone) + 0.69 × (other or unknown stone composition) + 1.23 × (intraoperative CBCT), where each variable is coded as 1 if present and 0 if absent, and the predicted probability is obtained by applying the logistic transformation p = 1/(1 + e^(−logit(p))), with single stone < 20 mm, calcium oxalate stone, and no intraoperative CBCT as the reference categories.

## Appendix B

**Table A1:** Comparison of characteristics between grade A (0 mm) stone-free cases after randomization versus cases with residual fragments > 0 mm after randomization.

Variable	Stone-Free, Grade A (*n* = 73)	Residual Fragments (*n* = 82)	*p*-Value
Baseline characteristics			
Age, years, median (IQR)	57 (49–66)	56.5 (45–66)	0.83 *
Female sex, *n* (%)	30 (41)	36 (44)	0.72 **
ASA class 3 or 4, *n* (%)	28 (38)	26 (32)	0.39 **
Left procedure side, *n* (%)	36 (49)	45 (55)	0.49 **
			
Weight characteristics			
BMI, kg/m^2^, median (IQR)	27.3 (24.0–30.9)	26.9 (23.8–31.1)	0.72 *
Abdominal circumference, cm, median (IQR)	100 (93–110)	98 (92–108)	0.46 *
Skin-to-stone distance, cm, median (IQR)	108 (90–118)	105 (88–120)	0.43 *
			
Preoperative profile			
Treatment < 1 year prior to PCNL, *n* (%)	17 (23)	21 (26)	0.74 **
Anatomical abnormalities, *n* (%)	16 (22)	20 (24)	0.72 **
Neurological abnormalities, *n* (%)	6 (8)	9 (11)	0.56 **
Preoperative hydronephrosis, *n* (%)	17 (23)	19 (23)	0.99 **
Preoperative drainage, *n* (%)	15 (21)	18 (22)	0.83 **
			
Radiological characteristics			
Maximum stone essence, HU, median (IQR)	1283 (1013–1635)	1127 (907–1481)	0.127 *
Stone volume, mm^3^, median (IQR)	360 (135–850)	580 (180–1933)	0.010 **
Intraoperative CBCT, *n* (%)	40 (55)	35 (43)	0.132 **
			
Stone size			<0.001 **
Single stone < 20 mm, *n* (%)	25 (34)	7 (8)	
Single stone > 20 mm, *n* (%)	6 (8)	6 (7)	
Multiple stones in ≤2 poles, *n* (%)	35 (48)	39 (48)	
Multiple stones in 3 poles, *n* (%)	2 (3)	12 (15)	
Partial or complete staghorn stone, *n* (%)	5 (7)	18 (22)	
			
Stone location			0.032 **
Pelvis, *n* (%)	13 (18)	8 (10)	
Upper pole, *n* (%)	4 (5)	2 (2)	
Middle pole, *n* (%)	4 (5)	1 (1)	
Lower pole, *n* (%)	26 (36)	22 (27)	
Multiple sites, *n* (%)	26 (36)	49 (60)	
			
Dominant stone composition			0.028 **
Calcium oxalate, *n* (%)	43 (59)	37 (45)	
Infectious, *n* (%)	15 (21)	35 (43)	
Uric Acid, *n* (%)	5 (7)	4 (5)	
Other or unknown composition, *n* (%)	10 (14)	6 (7)	

ASA = American Society of Anesthesiologists; BMI = body mass index; CBCT = cone-beam computed tomography; HU = Hounsfield units; IQR = interquartile range; PCNL = percutaneous nephrolithotomy. * Mann–Whitney U Test, ** Chi-squared test.

**Table A2:** Multivariate binary logistic regression results of predictors for a grade A (0 mm) stone-free status after percutaneous nephrolithotomy.

Variable	Model Coefficients	Odds Ratio	95% CI	*p*-Value
Intercept	1.03			
Single stone < 20 mm (reference class)				0.002
Single stone > 20 mm	−1.29	0.27	0.06–1.22	0.090
Multiple stones in ≤2 poles	−1.43	0.24	0.09–0.64	0.005
Multiple stones in 3 poles	−2.96	0.05	0.01–0.30	<0.001
Partial or complete staghorn stone	−2.45	0.09	0.02–0.35	<0.001
Calcium oxalate stone (reference class)				0.433
Infectious stone	−0.50	0.61	0.26–1.42	0.247
Uric acid stone	0.24	1.28	0.29–5.66	0.747
Other or unknown stone composition	0.46	1.58	0.46–5.46	0.471
No intraoperative CBCT (refence class)				0.057
Intraoperative CBCT	0.70	2.01	0.98–4.11	0.057

CBCT = cone-beam computed tomography; CI = confidence interval.

## Appendix C

Full Model Equation. The predicted probability of the outcome (grade A 0 mm stone-free rate) can be calculated using the following logistic regression model: logit(p) = 1.03 − 1.29 × (single stone > 20 mm) − 1.43 × (multiple stones in ≤2 poles) − 2.96 × (multiple stones in 3 poles) − 2.45 × (partial or complete staghorn stone) − 0.50 × (infectious stone) + 0.24 × (uric acid stone) + 0.46 × (other or unknown stone composition) + 0.70 × (intraoperative CBCT), where each variable is coded as 1 if present and 0 if absent, and the predicted probability is obtained using p = 1/(1 + e^(−logit(p))), with single stone < 20 mm, calcium oxalate stone, and no intraoperative CBCT as the reference categories.

## References

[1] Roy O.P., Angle J.F., Jenkins A.D., Schenkman N.S. (2012). Cone beam computed tomography for percutaneous nephrolithotomy: Initial evaluation of a new technology. J. Endourol..

[2] Nevo A., Holland R., Schreter E., Gilad R., Baniel J., Cohen A., Lifshitz D.A. (2018). How Reliable Is the Intraoperative Assessment of Residual Fragments During Percutaneous Nephrolithotomy? A Prospective Study. J. Endourol..

[3] Harraz A.M., Osman Y., El-Nahas A.R., Elsawy A.A., Fakhreldin I., Mahmoud O., El-Assmy A., Shokeir A.A. (2017). Residual stones after percutaneous nephrolithotomy: Comparison of intraoperative assessment and postoperative non-contrast computerized tomography. World J. Urol..

[4] Hartung F.O., Muller K.J., Herrmann J., Grune B., Michel M.S., Rassweiler-Seyfried M.C. (2023). Comparison of endoscopic versus CT assessment of stone-free status after percutaneous nephrolithotomy (PCNL). Urolithiasis.

[5] Sorensen M.D., Harper J.D., Borofsky M.S., Hameed T.A., Smoot K.J., Burke B.H., Levchak B.J., Williams J.C., Bailey M.R., Liu Z. (2022). Removal of Small, Asymptomatic Kidney Stones and Incidence of Relapse. N. Engl. J. Med..

[6] Roemeling S., Kingma R.A., Suijker C.A., Altobelli E., Bus M.T.J., Greuter M.J.W., Mahesh S.V.K., de Jong I.J. (2025). Intraoperative Cone Beam Computed Tomography Increases Single Procedure Stone-Free Rates in Percutaneous Nephrolithotomy: Results of a Randomized Controlled Trial. J. Endourol..

[7] Suijker C.A., Kingma R.A., van Ee R., Steffens E.N., Altobelli E., Bus M.T.J., de Jong I.J., Roemeling S. (2025). Longer-term effects of intraoperative cone-beam computed tomography in percutaneous nephrolithotomy: 18-month retrospective randomised controlled trial analysis. BJU Int..

[8] Patel P.M., Kandabarow A.M., Chuang E., McKenzie K., Druck A., Seffren C., Blanco-Martinez E., Capoccia E., Farooq A.V., Branch J. (2022). Using Intraoperative Portable CT Scan to Minimize Reintervention Rates in Percutaneous Nephrolithotomy: A Prospective Trial. J. Endourol..

[9] Van den Broeck T., Zhu X., Kusters A., Futterer J., Langenhuijsen J., d’Ancona F. (2021). Percutaneous Nephrolithotomy with Intraoperative Computed Tomography Scanning Improves Stone-Free Rates. J. Endourol..

[10] Glover X.G., Ballon-Landa E.C., Sawyer M.D. (2023). Ultralow-Dose Intraoperative Computed Tomography During Endoscopic Stone Surgery: A Quality Improvement Project. J. Endourol..

[11] Sauerbrei W. (1999). The use of resampling methods to simplify regression models in medical statistics. J. R. Stat. Soc..

[12] Royston P., Moons K.G., Altman D.G., Vergouwe Y. (2009). Prognosis and prognostic research: Developing a prognostic model. BMJ.

[13] van Geloven N., Keogh R.H., van Amsterdam W., Cina G., Krijthe J.H., Peek N., Luijken K., Magliacane S., Morzywolek P., van Ommen T. (2025). The Risks of Risk Assessment: Causal Blind Spots When Using Prediction Models for Treatment Decisions. Ann. Intern. Med..

[14] Kingma R.A., Mors R., Bus M.T.J., Altobelli E., de Jong I.J., Roemeling S. (2024). Cone Beam Computed Tomography-Assisted Percutaneous Nephrolithotomy in a Hybrid Operating Room: Optimization of Patient Selection. J. Endourol..

[15] Smith A., Averch T.D., Shahrour K., Opondo D., Daels F.P., Labate G., Turna B., de la Rosette J.J., Group C.P.S. (2013). A nephrolithometric nomogram to predict treatment success of percutaneous nephrolithotomy. J. Urol..

[16] Okhunov Z., Friedlander J.I., George A.K., Duty B.D., Moreira D.M., Srinivasan A.K., Hillelsohn J., Smith A.D., Okeke Z.S.T.O.N.E. (2013). nephrolithometry: Novel surgical classification system for kidney calculi. Urology.

[17] Zhu Z., Wang S., Xi Q., Bai J., Yu X., Liu J. (2011). Logistic regression model for predicting stone-free rate after minimally invasive percutaneous nephrolithotomy. Urology.

[18] Shahrour K., Tomaszewski J., Ortiz T., Scott E., Sternberg K.M., Jackman S.V., Averch T.D. (2012). Predictors of immediate postoperative outcome of single-tract percutaneous nephrolithotomy. Urology.

[19] Turna B., Umul M., Demiryoguran S., Altay B., Nazli O. (2007). How do increasing stone surface area and stone configuration affect overall outcome of percutaneous nephrolithotomy?. J. Endourol..

[20] Thomas K., Smith N.C., Hegarty N., Glass J.M. (2011). The Guy’s stone score--grading the complexity of percutaneous nephrolithotomy procedures. Urology.

[21] Lepine H.L., Vicentini F.C., Mazzucchi E., Molina W.R., Marchini G.S., Torricelli F.C., Batagello C.A., Danilovic A., Nahas W.C. (2024). Intraoperative computed tomography for detection of residual stones in endourology procedures: Systematic review and meta-analysis. Int. Braz. J. Urol..

[22] Osman M.M., Alfano Y., Kamp S., Haecker A., Alken P., Michel M.S., Knoll T. (2005). 5-year-follow-up of patients with clinically insignificant residual fragments after extracorporeal shockwave lithotripsy. Eur. Urol..

[23] Altunrende F., Tefekli A., Stein R.J., Autorino R., Yuruk E., Laydner H., Binbay M., Muslumanoglu A.Y. (2011). Clinically insignificant residual fragments after percutaneous nephrolithotomy: Medium-term follow-up. J. Endourol..

[24] Raman J.D., Bagrodia A., Gupta A., Bensalah K., Cadeddu J.A., Lotan Y., Pearle M.S. (2009). Natural history of residual fragments following percutaneous nephrostolithotomy. J. Urol..

[25] Osman Y., Harraz A.M., El-Nahas A.R., Awad B., El-Tabey N., Shebel H., Shoma A.M., Eraky I., El-Kenawy M. (2013). Clinically insignificant residual fragments: An acceptable term in the computed tomography era?. Urology.

